# Giant isolated intracardiac thrombus causing right ventricular outflow tract obstruction in an 85-year-old patient with renal cell carcinoma

**DOI:** 10.1093/omcr/omab066

**Published:** 2021-08-13

**Authors:** Georgios S Papaetis, Petros P Mavrommatis, Antreas D Antreou, Stylianos A Karvounaris

**Affiliations:** 1 Internal Medicine and Diabetes Clinic, Paphos, Cyprus; 2 CDA College, Paphos, Cyprus; 3 Cardiac Care Centre, Paphos, Cyprus; 4 Department of Radiology, Evangelismos Hospital, Paphos, Cyprus; 5 Department of Cardiology, Evangelismos Hospital, Paphos, Cyprus

**Figure 1:**
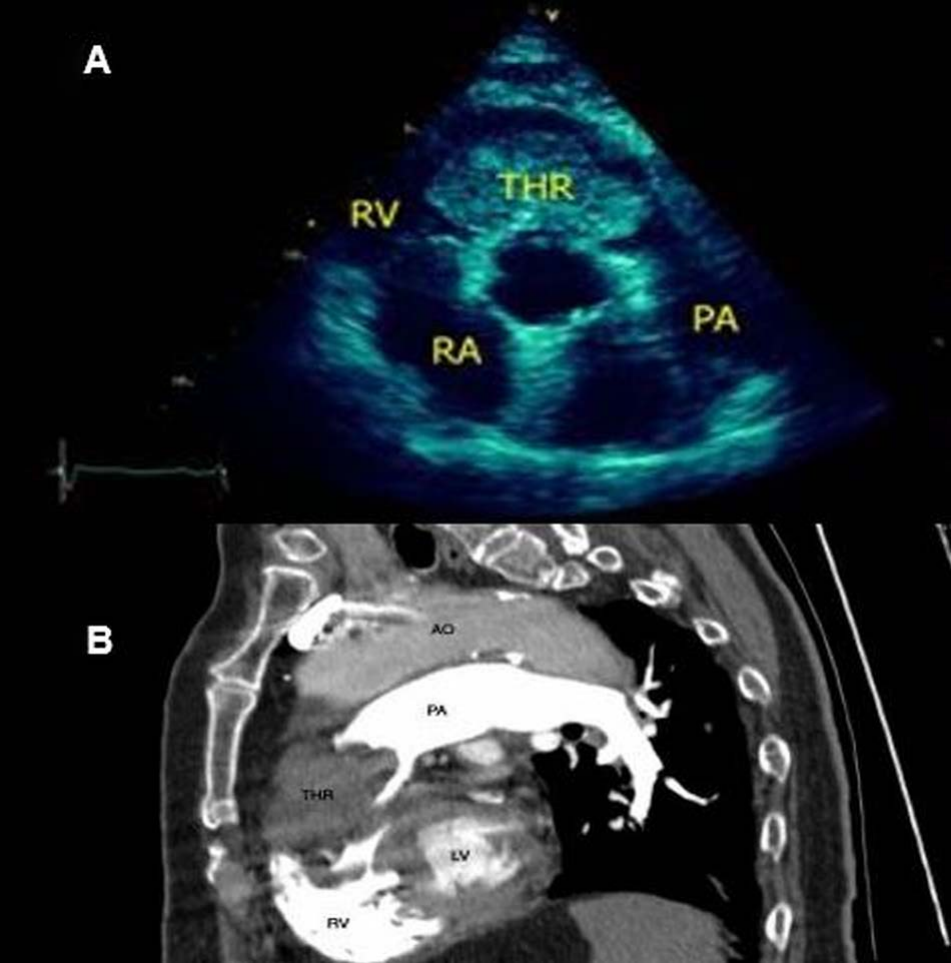
(A) TTE-SAX showing a giant echogenic mass in the RV obstructing the outflow track; (B) CTPA revealed a giant mass in the main PA extending into the left PA, with no evidence of vascularization, suggesting a thrombus.

An 85-year-old female presented complaining of acute dyspnoea and chest pain for the last 4 hours. Her past medical history was stage II renal cell carcinoma (RCC). The patient was dyspneic and afebrile. Her observations were a respiratory rate of 24, blood pressure of 95/60, oxygen saturations of 85% (corrected with 50% venturi mask) and a heart rate of 86 (after treating with a beta-blocker). The admission ‘electrocardiogram’ showed sinus rhythm, first degree atrioventricular block and supraventricular extrasystoles. Lung auscultation was normal without any signs of left heart failure. Transthoracic echocardiogram (TTE) revealed a giant mobile irregularly shaped echogenic mass in the right ventricle (RV), measuring 35 × 42 mm which was obstructing the right ventricular outflow tract (RVOT) (Fig. 1A). CT pulmonary angiography confirmed the presence of a huge mass (area: 18.575 cm^2^) in the main pulmonary artery obstructing the RVOT, with no evidence of vascularization, suggesting RVOT thrombus (Fig. 1B). Emboli in the pulmonary vessels were not found. No thrombosis was found in the peripheral systemic veins. The patient was given systemic thrombolysis but unfortunately, she passed away 3 days after.

Venous thromboembolic events are common and serious co-morbidities in older patients with RCC, however intracardial thrombi are extremely rare [1]. To our knowledge this is the third published report of RVOT obstruction from a massive thrombus in a patient with RCC and the first in an older patient. The other two cases were published in 2016 and 2018 in patients aged 62 and 51 years old, respectively [2, 3]. The detection of an isolated giant RVOT thrombus is an uncommon event. It has been mainly described in patients with arrhythmogenic RV dysplasia and autoimmune diseases (such as antiphospholipid antibody syndrome and Bechet disease) [4, 5]. In cancer patients isolated RVOT thrombi have been rarely described. Physicians should be vigilant for this rare complication in this clinical setting. Immediate thrombolysis or embolectomy should be considered as possible options [6]. Specific guidelines for the management of intracardiac thrombi should be established.
